# Property value estimation for inhaled therapeutic binary gas mixtures: He, Xe, N_2_O, and N_2 _with O_2_

**DOI:** 10.1186/2045-9912-1-28

**Published:** 2011-12-06

**Authors:** Ira Katz, Georges Caillibotte, Andrew R Martin, Philippe Arpentinier

**Affiliations:** 1 Medical Gases Group, Air Liquide Santé International, Centre de Recherche Claude-Delorme, Jouy-en-Josas, France; 2 Department of Mechanical Engineering, Lafayette College, Easton, PA, 18042, USA; 3 Scientific Direction, Air Liquide Research and Development, Centre de Recherche Claude-Delorme, Jouy-en-Josas, France

## Abstract

**Background:**

The property values of therapeutic gas mixtures are important in designing devices, defining delivery parameters, and in understanding the therapeutic effects. In the medical related literature the vast majority of articles related to gas mixtures report property values only for the pure substances or estimates based on concentration weighted averages. However, if the molecular size or structures of the component gases are very different a more accurate estimate should be considered.

**Findings:**

In this paper estimates based on kinetic theory are provided of density, viscosity, mean free path, thermal conductivity, specific heat at constant pressure, and diffusivity over a range of concentrations of He-O_2_, Xe-O_2_, N_2_O-O_2 _and N_2_-O_2 _mixtures at room (or normal) and body temperature, 20 and 37°C, respectively and at atmospheric pressure.

**Conclusions:**

Property value estimations have been provided for therapeutic gas mixtures and compared to experimental values obtained from the literature where possible.

## Introduction

Inhaled therapeutic gases in use today include helium (He) for respiratory treatments, and xenon (Xe) and nitrous oxide (N_2_O) for anesthesia. For clinical applications these gases are used in the form of mixtures with oxygen in a range of concentrations (typically starting from 20% oxygen (O_2_) concentration by volume, which is equivalent to a mole fraction of 0.20) so as to maintain adequate oxygenation. Other gases, such as nitric oxide (NO) for pulmonary vascular dilation, are used only in trace amounts.

The property values of therapeutic gas mixtures are important in designing devices, defining delivery parameters, and in understanding the therapeutic effects. Properties of interest include density, viscosity, mean free path, thermal conductivity, specific heat, and diffusivity. In the medical literature the vast majority of articles related to gas mixtures report property values only for the pure substances or estimates based on (volume or molar) concentration weighted averages [1-7]. However, if the molecular size or structures of the component gases are very different a more accurate estimate could be considered [8-10]. For this reason property values of helium and xenon mixtures should be considered for more accurate estimation.

Starting with kinetic theory for molecules treated as hard spheres as a basis, a rich literature has developed regarding the modeling of property values based on first principles and increasing complexity of the molecular interactions; in particular, the attraction and repulsion of molecules as first formulated by Chapman and Enskog [8,9]. The empirically determined Lennard-Jones potential energy function has proved to be a good model for many applications. Extensive measurements of the viscosity of gases using oscillating-disk viscometry have primarily been published by Kestin and his colleagues [11-16]. Other equilibrium and transport properties have been extrapolated from the viscosity measurements using the models described above [8,9]. There also exists limited thermal conductivity data measured using a hot wire method [17].

The objective of this short communication is to give a straightforward reference to the applied scientist, engineer, and medical personnel who perform research with therapeutic gas mixtures. We anticipate that this information will assist both in the design and interpretation of experiments. Estimates of density, viscosity, mean free path, thermal conductivity, specific heat at constant pressure, and diffusivity are provided over a range of concentrations of He-O_2_, Xe-O_2_, and N_2_O-O_2 _mixtures at room (or normal) and body temperature, 20 and 37°C, respectively and at atmospheric pressure; based on kinetic theory and compared to experimental values obtained from the literature where it is possible. For further comparison N_2_-O_2 _mixtures will be included because this mixture makes up the composition of medical air.

## Methods

### Density

All of the mixtures can be evaluated as ideal gases under the conditions considered. As such the density is based on the state equation as,

(1) $$\rho_{mix}=\frac{p}{R_{mix}T}$$

where ρ_mix _is the mixture density, p is the pressure, T is the absolute temperature and R_mix _is the gas constant defined for the mixture as

(2) $$R_{mix}=\frac{R_{univ}}{X_{i}MW_{i}+\left(1-X_{i}\right)32}$$

In Equation (2) R_univ _is the universal gas constant, X_i _is the mole fraction of the pure gas component, and MW_i _is the molecular weight of the pure gas component (32 is the molecular weight for oxygen). The units of R_mix _depends on the value chosen for R_univ _(e.g., 8314 N-m/kgmol-K).

### Viscosity

For viscosity we use a semi-empirical method by Wilke [8] that extends the model for collisions between hard spheres to mixtures.

(3a) $$\mu_{mix}=\frac{X_{i}\mu_{i}}{X_{i}+\left(1-X_{i}\right)\phi_{i-O_{2}}}+\frac{\left(1-X_{i}\right)\mu_{O_{2}}}{X_{i}\phi_{O_{2}-i}+\left(1-X_{i}\right)}$$

(3b) $$\phi_{i-O_{2}}=\frac{{\left[1+\sqrt{\frac{\mu_{i}}{\mu_{O_{2}}}}{\left(\frac{32}{MW_{i}}\right)}^{1∕4}\right]}^{2}}{\sqrt{8\left(1+\frac{MW_{i}}{32}\right)}}$$

(3c) $$\phi_{O_{2}-i}=\frac{{\left[1+\sqrt{\frac{\mu_{O_{2}}}{\mu_{i}}}{\left(\frac{MW_{i}}{32}\right)}^{1∕4}\right]}^{2}}{\sqrt{8\left(1+\frac{32}{MW_{i}}\right)}}$$

*μ_i _*and $\mu_{O_{2}}$are the viscosities of the pure gas component and oxygen, respectively. The pure gas viscosity estimates are based on the Lennard-Jones empirical function for the potential:

(4) $$\phi \left(r\right)=4\varepsilon \left[{\left(\frac{\sigma }{r}\right)}^{12}-{\left(\frac{\sigma }{r}\right)}^{6}\right]$$

where r is the distance between the molecules, *ε *is a characteristic energy of the interaction between molecules and σ is a characteristic diameter, or collision diameter. Equation (5) is a viscosity formula based on the Lennard-Jones parameters in units of kg/s-m derived for monatomic gases that has also been shown to work well for polyatomic gases [8],

(5) $$\mu_{i}=0.26693x10^{-5}\frac{\sqrt{MW_{i}T}}{\sigma^{2}{\text{Ω}}_{\mu }}$$

where Ω*_μ _*is a function of *ε*. Lennard-Jones parameters are tabulated for common gases [8,9] and for the gases herein in Table 1.

**Table 1:** Molecular parameters and Lennard-Jones potential parameters for the pure gas components [9].

Gas	MW	R (J/kg-K)	σ (Å)	ε/κ (°K)	Ωμ at 20°C	Ωμ at 37°C	Atomic Diffusion Volume (Σv)
**He**	4.003	2076.9	2.551	10.22	0.7061	0.7004	2.67

**Xe**	131.3	63.3	4.047	231.	1.4140	1.3798	32.7

**N_2_O**	44.02	188.9	3.828	232.4	1.4190	1.3846	35.9

**N_2_**	28.02	296.7	3.798	71.4	0.9697	0.9535	18.5

**O_2_**	32.00	259.8	3.467	106.7	1.0635	1.047	16.3

Values for Ω have been interpolated from Table B-2 in Bird et al. [8]. κ is the Boltzmann constant.

### Mean Free Path

The estimation of mean free path is based on the Chapman-Enskog formulation for hard spheres [18], where the mixture viscosity and density account for the interactions of the different molecules:

(6) $$\lambda_{mix}=\frac{16\mu_{mix}}{5\rho_{mix}\sqrt{2\pi R_{mix}T}}$$

The input values are obtained from Equations 1-3.

### Specific Heat at Constant Pressure

The specific heat at constant pressure (on a per unit mass basis) for all of the mixtures can be evaluated assuming ideal gas behavior and therefore the specific heat curve is a linear function of the mass fraction, though nonlinear in terms of the mole fraction

(7) $${c_{p}}_{mix}=\frac{X_{i}\rho_{i}}{\rho_{mix}}{c_{p}}_{i}+\frac{\left(1-X_{i}\right)\rho_{O_{2}}}{\rho_{mix}}{c_{p}}_{O_{2}}$$

where ${c_{p}}_{mix}$ and ${c_{p}}_{i}$ are the specific heats of the mixture and of the pure gas component, respectively. The pure gas values for the monatomic gases are based on the theoretical value $c_{p_{i}}=\frac{2.5R_{univ}}{MW_{i}}$ The polyatomic estimates are based on empirically derived 4^th ^order polynomials in temperature found in Poling et al. [9].

### Thermal Conductivity

Thermal conductivity is treated in an analogous manner to viscosity, where Equation (8a) is equivalent to Equation (3a) and the coefficients are exactly the same based on the pure gas viscosity values.

(8a) $$\mu_{mix}=\frac{X_{i}k_{i}}{X_{i}+\left(1-X_{i}\right)\phi_{i-O_{2}}}+\frac{\left(1-X_{i}\right)k_{O_{2}}}{X_{i}\phi_{O_{2}-i}+\left(1-X_{i}\right)}$$

(8b) $$\phi_{i-O_{2}}=\frac{{\left[1+\sqrt{\frac{\mu_{i}}{\mu_{O_{2}}}}{\left(\frac{32}{MW_{i}}\right)}^{1∕4}\right]}^{2}}{\sqrt{8\left(1+\frac{MW_{i}}{32}\right)}}$$

(8c) $$\phi_{O_{2}-i}=\frac{{\left[1+\sqrt{\frac{\mu_{O_{2}}}{\mu_{i}}}{\left(\frac{MW_{i}}{32}\right)}^{1∕4}\right]}^{2}}{\sqrt{8\left(1+\frac{32}{MW_{i}}\right)}}$$

The pure gas conductivity estimates are based on a modified Eucken approximation found in Poling et al. [9].

(9) $$k_{i}=\mu_{i}R_{i}\left(\frac{c_{p\;i}}{R_{i}}-1\right)\left(1.15+\frac{2.03}{\left(\frac{c_{p\;i}}{R_{i}}-1\right)}\right)$$

### Diffusivity

The self diffusivity for a binary system D_ij_, represents the movement of species i relative to the mixture, where D_ij _= D_ji_. The presentation here is based on the method of Fuller et al. given in Poling et al [9], which uses empirically obtained atomic diffusion volumes (Σv).

(10) $$D_{iO_{2}}=\frac{1.43x10^{-7}T^{1.75}}{\sqrt{2}p{\left(\frac{1}{MW_{i}}+\frac{1}{32}\right)}^{-1∕2}{\left[{\left(\Sigma_{v}\right)}_{i}^{1∕3}+{\left(16.3\right)}^{1∕3}\right]}^{2}}$$

In Equation (10) j always represents oxygen, the diffusivity is in m^2^/s, T is the temperature in degrees Kelvin, p is the pressure in bar and the atomic diffusion volumes are given in Table 1 for each gas. $D_{iO_{2}}$is almost independent of composition at low pressures so only a single value will be calculated for each binary gas pair [8].

Of much practical interest is the diffusivity of water vapor or carbon dioxide through the gas mixtures. Values are calculated for these mixtures based on Blanc's law [9].

(11) $$D_{mk}={\left(\frac{X_{j}}{D_{jk}}+\frac{X_{O_{2}}}{D_{O_{2}k}}\right)}^{-1}$$

Where m represents the therapeutic gas mixture considered, j represents the specific therapeutic gas, and k corresponds to H_2_O or CO_2_. The diffusion constants in Equation 11 of H_2_O or CO_2_ through the therapeutic gas or oxygen are calculated using Equation 10 with atomic diffusion volumes of 13.1 and 26.9 for H_2_O or CO_2_, respectively.

## Results

**Figure 1:**
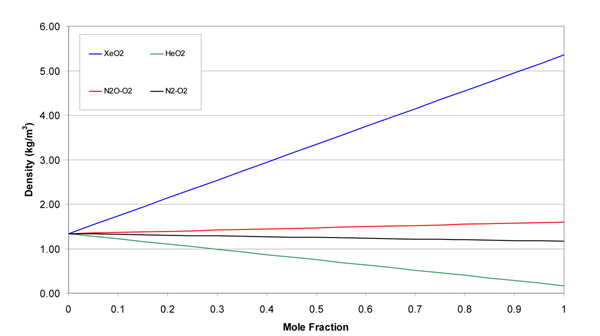
**Density of gas mixtures at 20°C and 1 atm**.

**Figure 2:**
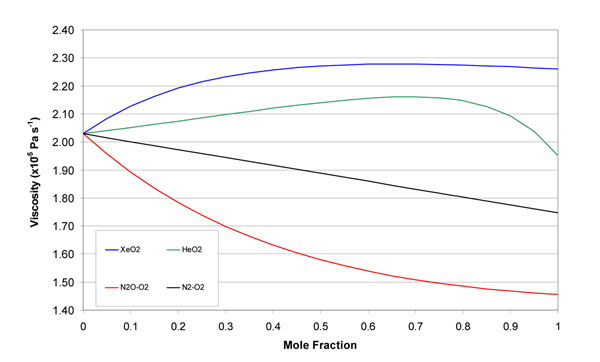
**Viscosity of gas mixtures at 20°C and 1 atm**.

**Figure 3:**
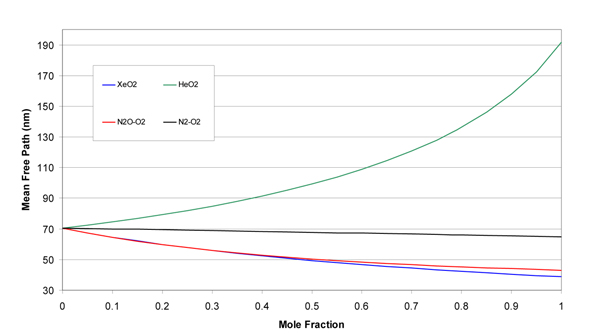
**Mean free path of gas mixtures at 20°C and 1 atm**.

**Figure 4:**
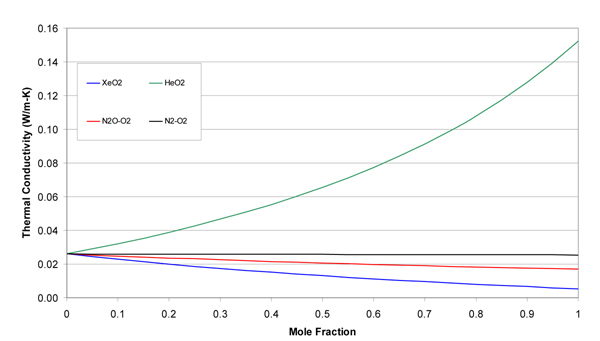
**Thermal conductivity of gas mixtures at 20°C and 1 atm**.

**Figure 5:**
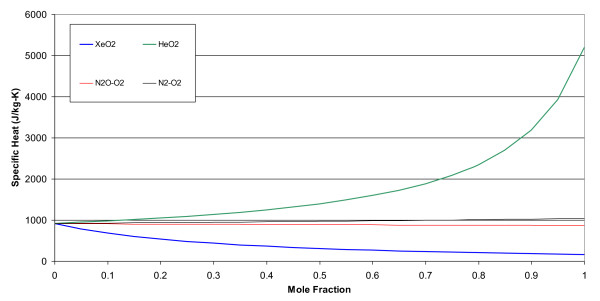
**Specific heat of gas mixtures at 20°C and 1 atm**.

The molecular weights, gas constants, Lennard-Jones parameters, and atomic diffusion volumes for the pure gases are given in Table 1. The mixture results are given in tabular and graphical forms. Tables 2, 3, 4, and 5 give the property values for He, Xe, N_2_O, and N_2 _with O_2 _mixtures, as a function of mole fraction at 20°C. Tables 6, 7, 8, and 9 are the analogous tables for 37°C. Table 10 gives binary diffusivities for the gas mixtures. Figures 1, 2, 3, 4, and 5 are plots of the 20°C data of density, viscosity, mean free path, thermal conductivity, and specific heat, respectively.

**Table 2:** He-O_2 _property values at 20°C and 1 atm.

He Mole Fraction	ρ (kg/m^3^)	μ × 10^5 ^(kg/s-m)	λ (n m)	k (W/m-K)	c_p _(J/kg-K)	$D_{H_{2}O}$**× 10^5 ^(m^2^/s)**	$D_{CO_{2}}$**× 10^5 ^(m^2^/s)**
**0**	1.330	2.029	70.561	0.026	917.5	2.551	1.573

**0.05**	1.272	2.040	72.547	0.029	945.5	2.641	1.632

**0.10**	1.214	2.051	74.673	0.032	976.1	2.739	1.695

**0.15**	1.156	2.063	76.954	0.035	1009.8	2.844	1.764

**0.20**	1.098	2.074	79.409	0.039	1047.1	2.957	1.838

**0.25**	1.039	2.086	82.057	0.043	1088.6	3.080	1.919

**0.30**	0.981	2.097	84.924	0.047	1135.0	3.214	2.007

**0.35**	0.923	2.109	88.038	0.051	1187.3	3.359	2.104

**0.40**	0.865	2.120	91.432	0.055	1246.6	3.519	2.210

**0.45**	0.807	2.131	95.148	0.060	1314.4	3.694	2.328

**0.50**	0.748	2.141	99.235	0.066	1392.8	3.888	2.459

**0.55**	0.690	2.149	103.751	0.071	1484.4	4.103	2.606

**0.60**	0.632	2.156	108.773	0.077	1592.9	4.343	2.772

**0.65**	0.574	2.161	114.393	0.084	1723.4	4.613	2.960

**0.70**	0.516	2.162	120.735	0.091	1883.3	4.919	3.175

**0.75**	0.457	2.158	127.959	0.099	2084.0	5.268	3.424

**0.78**	0.422	2.152	132.807	0.104	2230.9	5.503	3.593

**0.79**	0.411	2.150	134.522	0.106	2285.5	5.585	3.653

**0.80**	0.399	2.147	136.291	0.108	2343.2	5.671	3.715

**0.85**	0.341	2.127	146.059	0.117	2690.8	6.140	4.060

**0.90**	0.283	2.092	157.788	0.128	3181.5	6.694	4.477

**0.95**	0.225	2.037	172.409	0.139	3926.4	7.359	4.988

**1.0**	0.166	1.952	191.912	0.152	5192.4	8.169	5.632

**Table 3:** Xe-O_2 _property values at 20°C and 1 atm.

Xe Mole Fraction	ρ (kg/m^3^)	μ × 10^5 ^(kg/s-m)	λ (nm)	k (W/m-K)	c_p _(J/kg-K)	$D_{H_{2}O}$**× 10^5 ^(m^2^/s)**	$D_{CO_{2}}$**× 10^5 ^(m^2^/s)**
**0**	1.330	2.029	70.561	0.026	917.5	2.551	1.573

**0.05**	1.537	2.084	67.417	0.024	782.7	2.487	1.522

**0.10**	1.743	2.128	64.637	0.023	679.8	2.427	1.474

**0.15**	1.950	2.163	62.138	0.021	598.6	2.369	1.429

**0.20**	2.156	2.192	59.866	0.020	533.1	2.314	1.387

**0.25**	2.362	2.215	57.783	0.019	478.9	2.262	1.347

**0.30**	2.569	2.232	55.863	0.017	433.5	2.211	1.309

**0.35**	2.775	2.247	54.083	0.016	394.9	2.164	1.273

**0.40**	2.982	2.257	52.428	0.015	361.5	2.118	1.240

**0.45**	3.188	2.265	50.883	0.014	332.5	2.074	1.208

**0.50**	3.394	2.271	49.437	0.013	307.1	2.031	1.177

**0.55**	3.601	2.275	48.080	0.012	284.5	1.991	1.148

**0.60**	3.807	2.277	46.804	0.011	264.4	1.952	1.121

**0.65**	4.014	2.278	45.602	0.010	246.4	1.915	1.095

**0.70**	4.220	2.278	44.467	0.010	230.1	1.878	1.070

**0.75**	4.427	2.276	43.395	0.009	215.3	1.844	1.046

**0.80**	4.633	2.274	42.379	0.008	201.9	1.810	1.023

**0.85**	4.839	2.272	41.415	0.007	189.6	1.778	1.001

**0.90**	5.046	2.268	40.500	0.007	178.3	1.747	0.980

**0.95**	5.252	2.265	39.630	0.006	167.9	1.717	0.960

**1.0**	5.459	2.260	38.801	0.005	158.3	1.688	0.940

**Table 4:** N_2_O-O_2 _property values at 20°C and 1 atm.

N_2_O Mole Fraction	ρ (kg/m^3^)	μx10^5 ^(kg/s-m)	λ (nm)	k (W/m-K)	c_p _(J/kg-K)	$D_{H_{2}O}$**× 10^5 ^(m^2^/s)**	$D_{CO_{2}}$**× 10^5 ^(m^2^/s)**
**0**	1.330	2.029	70.561	0.026	917.5	2.551	1.573

**0.05**	1.355	1.956	67.394	0.025	914.3	2.500	1.542

**0.10**	1.380	1.892	64.577	0.025	911.1	2.451	1.511

**0.15**	1.405	1.835	62.065	0.024	908.1	2.404	1.482

**0.20**	1.430	1.784	59.820	0.024	905.2	2.358	1.454

**0.25**	1.455	1.739	57.810	0.023	902.4	2.315	1.426

**0.30**	1.480	1.699	56.005	0.023	899.7	2.273	1.400

**0.35**	1.505	1.664	54.383	0.022	897.1	2.232	1.375

**0.40**	1.530	1.632	52.923	0.022	894.6	2.193	1.351

**0.45**	1.555	1.605	51.605	0.021	892.1	2.156	1.327

**0.50**	1.580	1.580	50.414	0.021	889.7	2.119	1.305

**0.55**	1.605	1.559	49.337	0.020	887.4	2.084	1.283

**0.60**	1.630	1.540	48.361	0.020	885.2	2.050	1.262

**0.65**	1.655	1.523	47.475	0.019	883.0	2.017	1.241

**0.70**	1.680	1.508	46.670	0.019	880.9	1.985	1.221

**0.75**	1.705	1.496	45.938	0.019	878.9	1.954	1.202

**0.80**	1.730	1.485	45.271	0.018	876.9	1.924	1.184

**0.85**	1.755	1.475	44.663	0.018	875.0	1.895	1.166

**0.90**	1.780	1.467	44.108	0.018	873.1	1.866	1.148

**0.95**	1.805	1.461	43.601	0.017	871.3	1.839	1.131

**1.0**	1.830	1.455	43.137	0.017	869.6	1.812	1.115

**Table 5:** N_2_-O_2 _property values at 20°C and 1 atm.

N_2 _Mole Fraction	ρ (kg/m^3^)	μ × 10^5 ^(kg/s-m)	λ (nm)	k (W/m-K)	c_p _(J/kg-K)	$D_{H_{2}O}$**× 10^5 ^(m^2^/s)**	$D_{CO_{2}}$**× 10^5 ^(m^2^/s)**
**0**	1.330	2.029	70.561	0.026	917.5	2.551	1.573

**0.05**	1.322	2.015	70.289	0.026	922.8	2.548	1.573

**0.10**	1.314	2.001	70.016	0.026	928.2	2.546	1.573

**0.15**	1.306	1.987	69.743	0.026	933.7	2.543	1.573

**0.20**	1.297	1.973	69.468	0.026	939.3	2.541	1.573

**0.25**	1.289	1.959	69.192	0.026	944.9	2.538	1.573

**0.30**	1.281	1.945	68.915	0.026	950.6	2.536	1.573

**0.35**	1.272	1.931	68.637	0.026	956.3	2.533	1.573

**0.40**	1.264	1.916	68.358	0.026	962.2	2.531	1.574

**0.45**	1.256	1.902	68.077	0.026	968.1	2.529	1.574

**0.50**	1.248	1.888	67.796	0.026	974.1	2.526	1.574

**0.55**	1.239	1.874	67.513	0.026	980.2	2.524	1.574

**0.60**	1.231	1.860	67.230	0.026	986.3	2.521	1.574

**0.65**	1.223	1.846	66.945	0.026	992.6	2.519	1.574

**0.70**	1.215	1.832	66.659	0.026	998.9	2.516	1.574

**0.75**	1.206	1.818	66.371	0.026	1005.3	2.514	1.574

**0.78**	1.201	1.809	66.198	0.026	1009.2	2.513	1.574

**0.79**	1.200	1.806	66.141	0.026	1010.5	2.512	1.574

**0.80**	1.198	1.803	66.083	0.026	1011.8	2.512	1.574

**0.85**	1.190	1.789	65.793	0.026	1018.4	2.509	1.574

**0.90**	1.181	1.775	65.502	0.025	1025.1	2.507	1.574

**0.95**	1.173	1.761	65.210	0.025	1031.9	2.504	1.574

**1.0**	1.165	1.747	64.916	0.025	1038.7	2.502	1.574

**Table 6:** He-O_2 _property values at 37°C and 1 atm.

He Mole Fraction	ρ (kg/m^3^)	μ × 10^5 ^(kg/s-m)	λ (nm)	k (W/m-K)	c_p _(J/kg-K)	$D_{H_{2}O}$**× 10^5 ^(m^2^/s)**	$D_{CO_{2}}$**× 10^5 ^(m^2^/s)**
**0**	1.257	2.113	75.572	0.027	920.7	2.815	1.736

**0.05**	1.202	2.125	77.716	0.030	948.7	2.915	1.801

**0.10**	1.147	2.137	80.012	0.034	979.3	3.023	1.871

**0.15**	1.092	2.149	82.477	0.037	1013.0	3.139	1.947

**0.20**	1.037	2.162	85.131	0.041	1050.3	3.264	2.029

**0.25**	0.982	2.175	87.996	0.045	1091.7	3.400	2.118

**0.30**	0.927	2.188	91.101	0.049	1138.1	3.547	2.215

**0.35**	0.872	2.200	94.475	0.053	1190.3	3.708	2.322

**0.40**	0.817	2.213	98.157	0.058	1249.5	3.883	2.440

**0.45**	0.762	2.225	102.191	0.063	1317.3	4.077	2.570

**0.50**	0.707	2.236	106.633	0.069	1395.7	4.291	2.714

**0.55**	0.652	2.246	111.548	0.075	1487.2	4.528	2.876

**0.60**	0.597	2.255	117.019	0.081	1595.6	4.793	3.059

**0.65**	0.542	2.261	123.153	0.088	1726.0	5.091	3.266

**0.70**	0.487	2.264	130.085	0.096	1885.8	5.429	3.504

**0.75**	0.432	2.262	137.997	0.104	2086.3	5.814	3.779

**0.78**	0.399	2.258	143.316	0.110	2233.2	6.073	3.965

**0.79**	0.388	2.256	145.199	0.112	2287.7	6.165	4.032

**0.80**	0.377	2.254	147.142	0.114	2345.3	6.259	4.100

**0.85**	0.322	2.235	157.891	0.124	2692.7	6.777	4.481

**0.90**	0.267	2.202	170.832	0.135	3183.0	7.389	4.941

**0.95**	0.212	2.149	187.014	0.147	3927.4	8.122	5.505

**1.0**	0.157	2.064	208.666	0.161	5192.4	9.016	6.216

**Table 7:** Xe-O_2 _property values at 37°C and 1 atm.

Xe Mole Fraction	ρ (kg/m^3^)	μ × 10^5 ^(kg/s-m)	λ (nm)	k (W/m-K)	c_p _(J/kg-K)	$D_{H_{2}O}$**× 10^5 ^(m^2^/s)**	$D_{CO_{2}}$**× 10^5 ^(m^2^/s)**
**0**	1.257	2.113	75.572	0.027	920.7	2.815	1.736

**0.05**	1.453	2.173	72.306	0.025	785.3	2.745	1.680

**0.10**	1.648	2.221	69.409	0.024	682.0	2.678	1.627

**0.15**	1.843	2.261	66.798	0.022	600.5	2.615	1.577

**0.20**	2.038	2.293	64.418	0.021	534.7	2.554	1.530

**0.25**	2.233	2.319	62.231	0.020	480.3	2.496	1.486

**0.30**	2.428	2.339	60.210	0.018	434.7	2.441	1.445

**0.35**	2.623	2.356	58.334	0.017	395.9	2.388	1.405

**0.40**	2.818	2.369	56.586	0.016	362.4	2.337	1.368

**0.45**	3.013	2.378	54.951	0.015	333.3	2.289	1.333

**0.50**	3.208	2.386	53.419	0.014	307.7	2.242	1.299

**0.55**	3.404	2.391	51.980	0.013	285.1	2.197	1.267

**0.60**	3.599	2.395	50.625	0.012	264.9	2.154	1.237

**0.65**	3.794	2.397	49.346	0.011	246.7	2.113	1.208

**0.70**	3.989	2.397	48.138	0.010	230.4	2.073	1.180

**0.75**	4.184	2.397	46.995	0.009	215.6	2.035	1.154

**0.80**	4.379	2.396	45.911	0.008	202.1	1.998	1.129

**0.85**	4.574	2.393	44.882	0.008	189.7	1.962	1.105

**0.90**	4.769	2.391	43.904	0.007	178.4	1.928	1.081

**0.95**	4.964	2.387	42.973	0.006	168.0	1.895	1.059

**1.0**	5.159	2.384	42.086	0.006	158.3	1.863	1.038

**Table 8:** N_2_O-O_2 _property values at 37°C and 1 atm.

N_2_O Mole Fraction	ρ (kg/m^3^)	μ × 10^5 ^(kg/s-m)	λ (nm)	k (W/m-K)	c_p _(J/kg-K)	$D_{H_{2}O}$**× 10^5 ^(m^2^/s)**	$D_{CO_{2}}$**× 10^5 ^(m^2^/s)**
**0**	1.257	2.113	75.572	0.027	920.7	2.815	1.736

**0.05**	1.281	2.039	72.254	0.027	918.4	2.759	1.701

**0.10**	1.305	1.974	69.300	0.026	916.2	2.705	1.668

**0.15**	1.328	1.916	66.666	0.025	914.0	2.653	1.635

**0.20**	1.352	1.864	64.312	0.025	911.9	2.603	1.604

**0.25**	1.376	1.819	62.203	0.024	909.9	2.555	1.574

**0.30**	1.399	1.779	60.311	0.024	908.0	2.509	1.546

**0.35**	1.423	1.743	58.611	0.023	906.1	2.464	1.518

**0.40**	1.446	1.712	57.080	0.023	904.3	2.421	1.491

**0.45**	1.470	1.684	55.700	0.022	902.5	2.379	1.465

**0.50**	1.494	1.659	54.454	0.022	900.8	2.339	1.440

**0.55**	1.517	1.638	53.327	0.021	899.1	2.300	1.416

**0.60**	1.541	1.619	52.307	0.021	897.5	2.262	1.393

**0.65**	1.564	1.602	51.382	0.021	896.0	2.226	1.370

**0.70**	1.588	1.588	50.543	0.020	894.5	2.190	1.348

**0.75**	1.612	1.576	49.780	0.020	893.0	2.156	1.327

**0.80**	1.635	1.565	49.087	0.020	891.6	2.123	1.306

**0.85**	1.659	1.556	48.455	0.019	890.2	2.091	1.286

**0.90**	1.683	1.549	47.880	0.019	888.9	2.060	1.267

**0.95**	1.706	1.542	47.355	0.019	887.6	2.030	1.248

**1.0**	1.730	1.537	46.876	0.018	886.3	2.000	1.230

**Table 9:** N_2_-O_2 _property values at 37°C and 1 atm.

N_2 _Mole Fraction	ρ (kg/m^3^)	μ × 10^5 ^(kg/s-m)	λ (nm)	k (W/m-K)	c_p _(J/kg-K)	$D_{H_{2}O}$**× 10^5 ^(m^2^/s)**	$D_{CO_{2}}$**× 10^5 ^(m^2^/s)**
**0**	1.257	2.113	75.572	0.027	920.7	2.815	1.736

**0.05**	1.250	2.098	75.262	0.027	925.9	2.812	1.736

**0.10**	1.242	2.083	74.950	0.027	931.2	2.810	1.736

**0.15**	1.234	2.067	74.638	0.027	936.6	2.807	1.736

**0.20**	1.226	2.052	74.324	0.027	942.0	2.804	1.737

**0.25**	1.218	2.037	74.010	0.027	947.5	2.802	1.737

**0.30**	1.211	2.022	73.694	0.027	953.1	2.799	1.737

**0.35**	1.203	2.006	73.377	0.027	958.7	2.796	1.737

**0.40**	1.195	1.991	73.059	0.027	964.4	2.793	1.737

**0.45**	1.187	1.976	72.740	0.027	970.2	2.791	1.737

**0.50**	1.179	1.961	72.420	0.027	976.1	2.788	1.737

**0.55**	1.171	1.946	72.098	0.027	982.0	2.785	1.737

**0.60**	1.164	1.931	71.776	0.027	988.0	2.783	1.737

**0.65**	1.156	1.915	71.452	0.027	994.1	2.780	1.737

**0.70**	1.148	1.900	71.127	0.027	1000.3	2.777	1.737

**0.75**	1.140	1.885	70.801	0.027	1006.6	2.775	1.737

**0.78**	1.135	1.876	70.605	0.027	1010.4	2.773	1.737

**0.79**	1.134	1.873	70.539	0.027	1011.6	2.773	1.737

**0.80**	1.132	1.870	70.474	0.027	1012.9	2.772	1.737

**0.85**	1.124	1.855	70.145	0.026	1019.4	2.769	1.737

**0.90**	1.117	1.840	69.815	0.026	1025.9	2.767	1.737

**0.95**	1.109	1.824	69.484	0.026	1032.5	2.764	1.737

**1.0**	1.101	1.809	69.152	0.026	1039.2	2.761	1.737

**Table 10:** Binary diffusivities at 1 atm.

Gas	$D_{iO_{2}}$**× 10^5 ^(m^2^/s)**	
	**20°C**	**37°C**

**He-O_2_**	7.142	7.883

**Xe-O_2_**	1.243	1.372

**N_2_O-O_2_**	1.415	1.561

**N_2_-O_2_**	1.999	2.206

## Discussion

In this paper thermophysical property values have been presented for inhaled therapeutic binary gas mixtures. Pure substance values at 20°C and 37°C and mixing formulas based on kinetic theory were used to estimate the mixture values. The approach was to use relatively simple estimates for nonpolar gases [8]. That is, more complex intermolecular interactions that occur, for example, at high pressure, were not included.

Whereas many therapeutic gases (e.g.; CO and NO) are used at trace concentrations such that property values of the bulk mixture are essentially equivalent to those of air, mixtures considered herein have significantly different properties than air which change as a function of component concentration. Mechanical property values of density and viscosity are fundamental to the understanding of gas transport and airway resistance. The thermal properties of conductivity and capacity are necessary to accurately predict how gas treatments will affect the temperature and humidity of the respiratory tract. They also will influence the thermodynamic interaction of inhaled aerosols with the gas, and thus the deposition distribution which is particularly relevant for helium-oxygen mixtures. Diffusion is a key mode of gas transport deep in the lung potentially affecting exchange with the blood.

Bird et al. [8] note that the concept of the mean free path is applicable only if there are no long range forces associated with the hard sphere kinetic theory models. For this reason it is not typically an element of modern kinetic theory. Nevertheless, it is an important parameter in modeling the interaction of aerosols and gases [19], and thus for combination therapies involving aerosols and gas mixtures. In contrast to the scheme employed by Loeb [20], the estimation method employed here does not directly take into account the molecular collisions. However, Equation (6) for the mean free path does account for the collisions of different molecules through the mixture viscosity. As the utility of this parameter in aerosol mechanics is to estimate a reduced drag on small particles where their size is comparable to the mean free path, this approach would appear to be self consistent.

**Figure 6:**
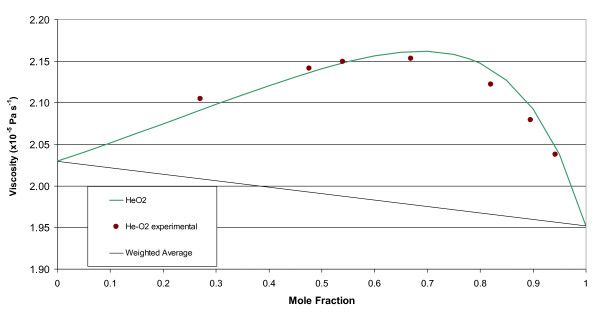
**Viscosity of He-O_2 _mixtures using Equation (3), based on a weighted average of the molar fractions and from experimental measurements **[14].

A comparison of estimated data based on Equation (3) to experimental data for the viscosity at 20°C of helium-oxygen mixtures [14] is shown in Figure 6, along with the linear curve representing the concentration weighted average. The maximum relative difference of 0.9% between the theory and experiment occurs at X_He _= 0.82. For the concentration weighted average value the maximum relative error of 7.9% occurs at X_He _= 0.67.

**Figure 7:**
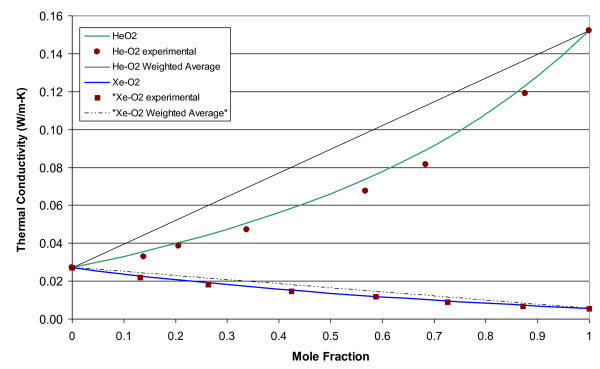
**Thermal conductivity at 30°C for He-O_2 _and Xe-O_2 _mixtures using Equation (8), based on a weighted average of the molar fractions and from experimental measurements **[17].

Figure 7 shows comparisons of experimental thermal conductivity values [17] for helium-oxygen and xenon-oxygen mixtures at 30°C compared to theoretical values calculated using Equation (8). The maximum relative differences between the theory and experiment are 4.2% at X_He _= 0.68 and 4.7% at X_Xe _= 0.27, respectively.

Table 11 shows a good agreement between experimental data for binary diffusivity of He-O_2 _and Xe-O_2 _[14,21] with theoretical data calculated using Equation (10). For the diffusivity of water vapor or carbon dioxide, the simplifying assumption leading to Blanc's law is for a *trace *component diffusing into a *homogeneous*, binary mixture. A quantitative definition of trace for the applicability of this assumption was not found. However, experiments testing diffusion of He, CO and SF_6 _through gas mixtures similar to alveolar gas (14% O_2_, 6% CO_2 _and 80% N_2_) did not show significant departures from values predicted on the basis of binary diffusion coefficient values weighted according to fractional concentrations [22] in agreement with Blanc's law. These experiments were performed with test gas concentrations varying from 0 to 10% suggesting Blanc's law would be appropriate for typical applications of the gases considered herein.

**Table 11:** Comparison of experimental and theoretical binary diffusivities based on Equation (10).

	$D_{i-O_{2}}$**× 10^5 ^(m^2^/s)**		
**T (K)**	**Experimental**	**Theoretical**	**Percent Difference**

**He-O_2_**			

**298 **[14]	7.06	7.357	4.21

**300 **[21]	7.441	7.437	0.05

**Xe-O_2_**			

**280 **[21]	1.147	1.128	1.68

**290 **[21]	1.220	1.202	1.50

**300 **[21]	1.295	1.279	1.25

**310 **[21]	1.371	1.357	1.03

**320 **[21]	1.449	1.438	0.76

In conclusion, the methods presented above allow accurate estimation of thermophysical property values for inhaled therapeutic binary gas mixtures, including He-O_2_, Xe-O_2_, and N_2_O-O_2_, over a range of concentrations.

## Competing interests

The authors declare that they have no competing interests.

## Authors' contributions

All of the authors have read and approved the final manuscript.

IK determined the appropriate models, wrote the software to implement the models and drafted the manuscript.

GC provided assistance with determining the models, implementing the software and edited the manuscript.

AM provided assistance with determining the models, implementing the software and edited the manuscript.

PA provided experimental data from the literature and edited the manuscript.
